# Redefining prostate cancer risk stratification: a pioneering strategy to estimate outcome based on Ki67 immunoscoring

**DOI:** 10.1186/s40364-024-00627-4

**Published:** 2024-08-01

**Authors:** Ângela Albuquerque-Castro, Catarina Macedo-Silva, Rúben Oliveira-Sousa, Vera Constâncio, João Lobo, Isa Carneiro, Rui Henrique, Carmen Jerónimo

**Affiliations:** 1 Cancer Biology & Epigenetics Group, Research Center of IPO Porto (CI-IPOP)/ CI-IPOP@ RISE (Health Research Network), Portuguese Oncology Institute of Porto (IPO-Porto)/Porto Comprehensive Cancer Center Raquel Seruca (Porto.CCC), R. Dr. António Bernardino de Almeida, 4200-072 Porto, Portugal; 2 Masters’ in Oncology, ICBAS-School of Medicine and Biomedical Sciences, University of Porto (ICBAS-UP), Rua Jorge Viterbo Ferreira 228, 4050-513 Porto, Portugal; 3 Doctoral Program in Biomedical Sciences, ICBAS-School of Medicine and Biomedical Sciences, ICBAS-UP), University of Porto, University of Porto (ICBAS-UP), Rua Jorge Viterbo Ferreira 228, 4050-513 Porto, Portugal; 4 Department of Pathology, Portuguese Oncology Institute of Porto (IPO Porto) / Porto Comprehensive Cancer Center Raquel Seruca (Porto.CCC), Research Center-LAB 3, F Bdg, 1st floor, Rua Dr António Bernardino de Almeida, Porto, 4200-072 Portugal; 5 Department of Pathology and Molecular Immunology, ICBAS-School of Medicine and Biomedical Sciences, University of Porto, Rua Jorge de Viterbo Ferreira 228, 4050-313 Porto, Portugal

**Keywords:** Prostate cancer, Prognostic, Biochemical recurrence, Biomarker, Ki67, DNA methylation

## Abstract

Accurate prostate cancer (PCa) patient diagnosis and risk assessment are key to ensure the best outcome. Currently, low- and favorable intermediate-risk PCa patients may be offered AS due to the indolent nature of the disease. Nonetheless, deciding between active surveillance and curative-intent treatment remains an intricate task, as a subset of these patients may eventually progress, enduring poorer prognosis. Herein, we sought to construct risk calculators based on cancer biomarkers, enabling more accurate discrimination among patients which may benefit from active interventions.

Ki67 immunoscore, *GSTP1* and *KLF8* promoter methylation levels (^me^) were assessed in PCa tissues. Study endpoints included overall and biochemical recurrence-free (BCR) survival. Combination with relevant clinicopathological parameters allowed for construction of graphical calculating tools (nomograms).

Higher Ki67 index correlated with worse BCR-free survival, whereas higher *KLF8*^*me*^ levels were associated with improved overall survival, especially in patients with lower-grade tumors. *GSTP1*^*me*^ levels had no prognostic value. Among prognostic models tested, a BCR-risk calculator – ProstARK (including Ki67 and clinicopathologic parameters) – disclosed 79.17% specificity, 66.67% sensitivity, 55% positive predictive value, 86% negative predictive value, and 75.76% accuracy. Similar results were found using an independent PCa biopsy cohort, validating its prognostication ability.

Combining clinicopathologic features and Ki67 index into a risk calculator enables easy and accurate implementation of a novel PCa prognostication tool. This nomogram may be useful for a more accurate selection of patients for active surveillance protocols. Nonetheless, validation in a larger, multicentric, set of diagnostic PCa biopsies is mandatory for further confirmation of these results.

**Supplementary Information:**

The online version contains supplementary material available at 10.1186/s40364-024-00627-4.

To the editor,

Accurate prostate cancer (PCa) risk assessment is key to ensure the best outcome. Nearly 90% of PCa are diagnosed as organ-confined [1] making clinical decisions on the best therapeutic strategy challenging [2, 3]. For low- and favorable intermediate-risk PCa- grade group (GG) 1 and 2- active surveillance (AS) is frequently considered [2], although this is not risk-exempt considering that some patients ultimately experience disease progression [4]. Currently, patients in AS are monitored through frequent biopsies, without uniform guidelines, and are proposed for active treatment only when increased tumor grade or stage is detected [4, 5]. Because accurate prognostic biomarkers may assist clinicians in deciding the best strategy, we sought to investigate whether molecular [*GSTP1* and *KLF8* promoter hypermethylation (^me^)] and/or immunohistochemical (Ki67 immunoscore) biomarkers might discriminate among patients with low or high-risk of PCa progression, using prostatectomy-derived tissues with subsequent validation in diagnostic biopsies. Ki67 index is a marker of aggressiveness/recurrence in several cancers [6], however, in PCa is not yet the standard care [7]. DNA methylation-based biomarkers, like *GSTP1*^*me*^ and *KLF8*^me^ hold promise for cancer detection and prognostication [1], although *KLF8*^me^ needs further exploration [1, 8].

Although we and others have shown the value of *GSTP1*^*me*^ for PCa detection [8], its prognostic performance was rather limited in this study, only significantly differing between stage III and stage II PCa patients (supplementary figure S1A). *KLF8*^*me*^ levels significantly differed between higher and lower tumor stages and low *KLF8*^*me*^ and significantly associated with worse overall survival (OS) in GG1/2 PCa patients (Supplementary Figure S1B and S2A). Nonetheless, this has limited clinical significance because OS is mostly influenced by age.

Confirming Ki67 immunoscore as a promising PCa prognostic biomarker [9, 10], we found that it correlated with higher stage and GG, demonstrating the association between proliferation and tumor aggressiveness (Supplementary Figure S1C/D). Importantly, high Ki67 immunoscore significantly associated with shorter biochemical recurrence (BCR)-free survival (a surrogate for metastatic disease and mortality), but not with OS (*data not shown*), recapitulating similar findings in other tumor models [11]. Specifically, Ki67 score 2 and 3 PCa patients endured significantly lower BCR-free survival, both globally and considering GG1/2 patients only, respectively (Supplementary Figure S2B/C).

Then, we developed risk calculators, which predicted risk of death (model 1: death risk = -4.0594 + 1.0339*AGEcat + 1.7585*KLF8 + 0.8255*Tstage-0.5032*PSA) and of BCR (ProstARK/model 2: BCR risk = -4.5509–0.1447*AGEcat + 1.6921*Ki67 + 0.5982*Tstage + 0.9164*PSA) (Fig. 1A/B and Table S1) with an area under de curve (AUC) > 0.75 (Fig. 1C) for GG1/2 patients and the whole cohort (Table S2 and Supplementary Figure S3). The purpose of both calculators was to provide accurate risk assessment at diagnosis, particularly focusing on low-grade patients, whom are candidates for AS. Both calculators significantly discriminated low- *versus* high-risk of death/BCR patients, respectively, for GG1/2 patients and the whole cohort(Fig. 2A/B and Supplementary Figure S4A/B). Notably, ProstARK performance was encouraging: 79.17% specificity, 66.67% sensitivity, 55% positive predictive value (PPV), 86% negative predictive value (NPV) and 75.76% accuracy (Table S3).

**Fig. 1:**
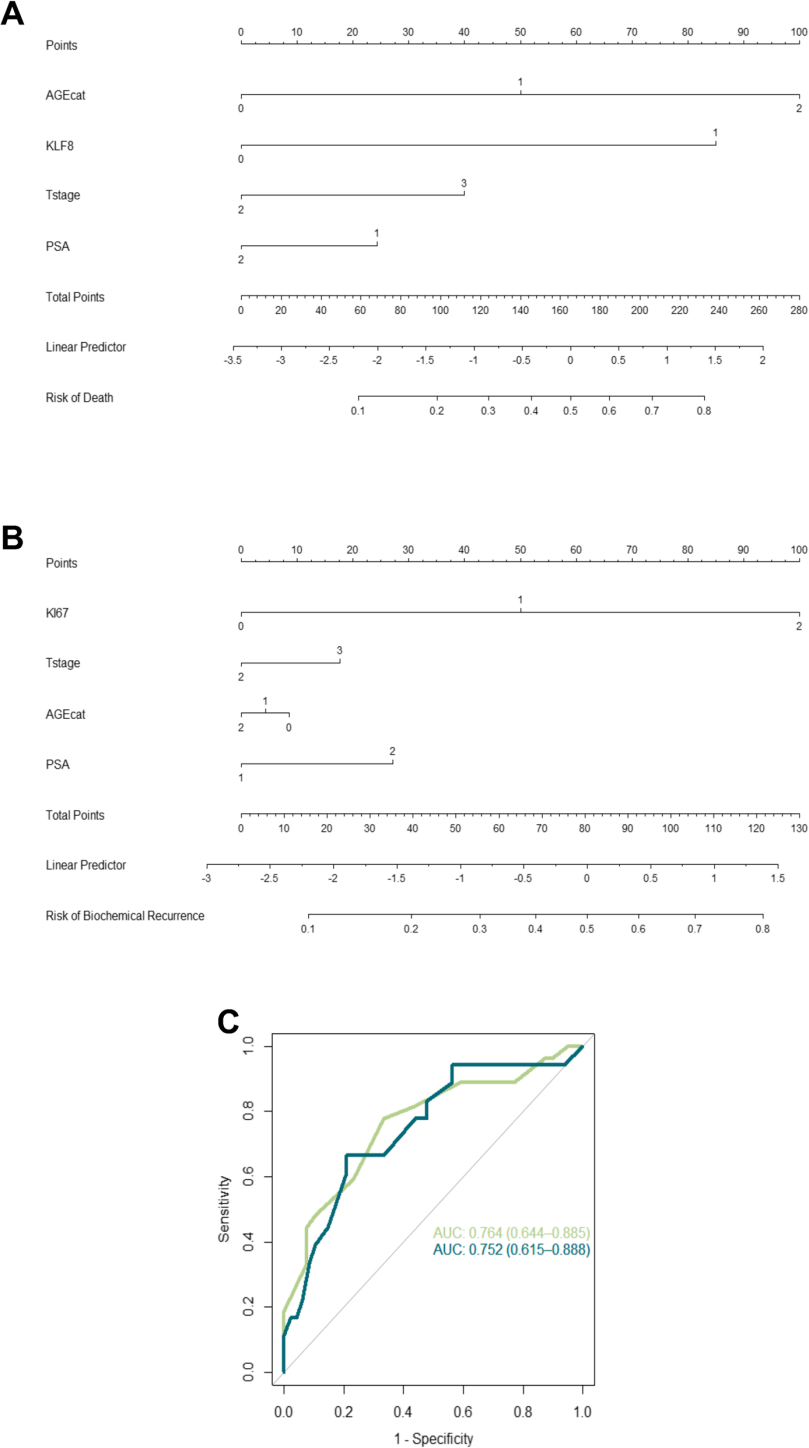
Graphical computation of a mathematical function integrating conventionally defined clinicopathological variables with new biomarkers. Nomogram representative of risk of death (**A**) and risk of biochemical-recurrence (**B**) calculators, for the Discovery Cohort GG1/2, with the relevant clinicopathological variables for risk stratification: age at diagnosis (0: ≤ 55, 1: 55–65, 2: > 65 years), clinical stage and PSA serum levels (1: < 10ng/mL, 2: > 10ng/mL). C- ROC curves to evaluate the performance of the nomogram models. *KLF8*^*me*^ categorization- 0: high methylation levels, 1: low methylation levels. Ki67 immunoexpression categorization- 0: < 5%, 1: 5–10%, 2: > 10% of positive staining. Green Curve: ROC curve for the Risk of Death calculator. Blue Curve: ROC curve for the Risk of Biochemical-recurrence calculator

Lastly, ProstARK performance was validated in an independent series of diagnostic prostate biopsies, simulating a risk assessment realistic scenario. Overall, AUC = 0.723 (95% CI: 0.571–0.875, Fig. 2C) was disclosed for GG1/2 PCa, slightly inferior to analysis of all the cases (Supplementary Figure S4C). Furthermore, ProstARK significantly discriminated low from high-risk patients (Fig. 2D and Supplementary Figure S4D).

**Fig. 2:**
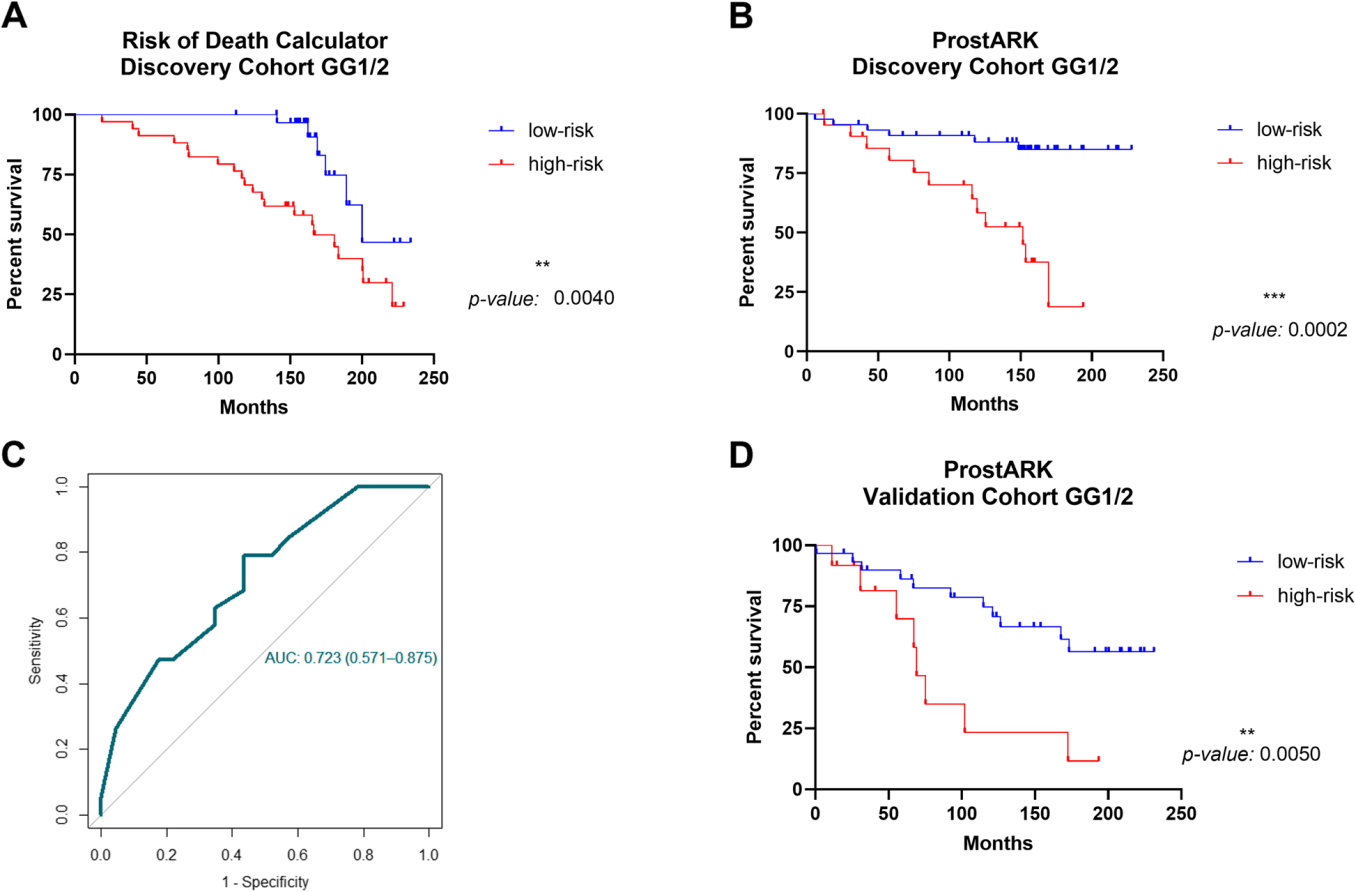
Risk Calculators evaluation in the Discovery and Validation Cohorts GG1/2. **A** Kaplan–Meier curve for overall survival based on the risk of death calculator, in the Discovery Cohort GG1/2. A -0.37 cut-off for the linear predictor translates into risk of death of 0.41. **B** Kaplan–Meier curves for the biochemical-recurrence free survival based on the ProstARK calculator, in Discovery Cohort GG1/2. A -0.82 cut-off for the linear predictor translates into recurrence/progression risk of 0.31. **C** ROC curve to evaluate the performance of the ProstARK, in Validation Cohort GG1/2 patients. **D** Kaplan–Meier curve for the biochemical-recurrence free survival based on the ProstARK calculator, in Validation Cohort GG1/2 patients. A -0.82 cut-off for the linear predictor translates into recurrence/progression risk of 0.31 in **D**)

Importantly, ProstARK high NPV suggests that it may assist in more accurately identifying patients benefiting from AS. It is tempting to speculate whether, in this scenario, ProstARK may better discriminate patients at risk for progression, despite unchanged grade or stage, to which active therapy might be offered. This may allow a reduction of subsequent, needless, biopsies with the refinement of the follow-up strategies for patients at higher risk for recurrence/progression.

Other genomic tests are currently available with the same purpose. Although Decipher® predicts adverse pathology (AUC = 0.65), it is less effective for AS [12]. Oncotype DX® has similar limitations (AUC = 0.68) [12], whereas Prolaris® predicts recurrence (AUC = 0.825), but at high cost [12]. ProstARK is cost-effective and accessible, unlike genomic tests, and leverages widely available equipment and know-how in pathology labs. Eventually, with the rise of Digital Pathology, Ki67 scoring may be further perfected.

In conclusion, the combination of clinicopathologic parameters and Ki67 into a risk calculator enables easy and accurate implementation of a novel PCa prognostication tool. A multicentric validation study using diagnostic PCa biopsies is planned and may include additional promising biomarkers.

## Supplementary Information

Supplementary Material 1.

## Data Availability

No datasets were generated or analysed during the current study.

## Abbreviations

PCa: Prostate cancer
AS: Active surveillance
OS: Overall survival
BCR: Biochemical recurrence
GG: Grade group
PSA: Prostate specific antigen
AUC: Area under the curve
PPV: Positive predictive value
NPV: Negative predictive value
