# Correction: Redefining prostate cancer risk stratification: a pioneering strategy to estimate outcome based on Ki67 immunoscoring

**DOI:** 10.1186/s40364-024-00670-1

**Published:** 2024-10-12

**Authors:** Ângela Albuquerque-Castro, Catarina Macedo-Silva, Rúben Oliveira-Sousa, Vera Constâncio, João Lobo, Isa Carneiro, Rui Henrique, Carmen Jerónimo

**Affiliations:** 1 Cancer Biology & Epigenetics Group, Research Center of IPO Porto (CI-IPOP)/ CI-IPOP@ RISE (Health Research Network), Portuguese Oncology Institute of Porto (IPO-Porto)/Porto Comprehensive Cancer Center Raquel Seruca (Porto.CCC), R. Dr. António Bernardino de Almeida, Porto, 4200-072 Portugal; 2 Masters’ in Oncology, ICBAS-School of Medicine and Biomedical Sciences, University of Porto (ICBAS-UP), Rua Jorge Viterbo Ferreira 228, Porto, 4050-513 Portugal; 3 Doctoral Program in Biomedical Sciences, ICBAS-School of Medicine and Biomedical Sciences, ICBAS-UP), University of Porto, University of Porto (ICBAS-UP), Rua Jorge Viterbo Ferreira 228, Porto, 4050-513 Portugal; 4 Department of Pathology, Portuguese Oncology Institute of Porto (IPO Porto)/Porto Comprehensive Cancer Center Raquel Seruca (Porto.CCC), Research Center-LAB 3, F Bdg, 1st floor, Rua Dr António Bernardino de Almeida, Porto, 4200-072 Portugal; 5 Department of Pathology and Molecular Immunology, ICBAS-School of Medicine and Biomedical Sciences, University of Porto, Rua Jorge de Viterbo Ferreira 228, Porto, 4050-313 Portugal

**Correction: Biomarker Research (2024) 12:75**

10.1186/s40364-024-00627-4

The authors mistakenly omitted a Competing Interest declaration in the original article; the correct Competing Interests declaration can be viewed ahead in this Correction article:

**Competing Interests**

There are no competing financial interests concerning the work described. The team has filed a patent application (20242005764083) for their groundbreaking method undersigned the “ProstARK calculator”- Prostate Assessment Risk with Ki67.

