# Supplementary material for: Redefining prostate cancer risk stratification: a pioneering strategy to estimate outcome based on Ki67 immunoscoring
Source: Biomark Res. 2024 Aug 1;12:75. doi: 10.1186/s40364-024-00627-4 (PMC11295892; doi:10.1186/s40364-024-00627-4)
Supplement: Supplementary file 1 — Supplementary Material 1. [file 40364_2024_627_MOESM1_ESM.docx]

Supplementary Materials for

Redefining prostate cancer risk stratification: a pioneering strategy to estimate outcome based on Ki67 immunoscoring.

Ângela Albuquerque-Castro; Catarina Macedo-Silva; Rúben Oliveira-Sousa; Vera Constâncio; João Lobo; Isa Carneiro; Rui Henrique; Carmen Jerónimo

Correspondence to: carmenjeronimo@ipoporto.min-saude.pt / [cljeronimo@icbas.up.pt](mailto:cljeronimo@icbas.up.pt)

**This file includes:**

Methodology details

Supplementary Figures S1 to S4 and respective captions

Tables S1 to S8 and respective captions

**Methods**

**Patients and samples**

Prostatectomy specimens (n=103), without prior treatment, were collected (2004-2008) at IPO Porto. Grade Group (GG) 1 and 2 patients were segregated as Discovery Cohort GG1/2. Table S4 summarizes clinicopathological data. Next, a validation series of 153 PCa diagnostic biopsies (Table S5), previously reported by us(1), was used to validate ProstARK performance.

**Immunohistochemistry (IHC)**

In the previous study by *João Lobo et al*. Ki67 immunoexpression was identified as the biomarker with the highest performance, capable of predicting disease recurrence at diagnosis in PCa patients with low/intermediate risk CAPRA scores, which are of particular interest for the current study. Although Ki-67 immunoscore has frequently been promoted as a valuable prognostic biomarker, it has not yet been integrated into standard care. Thus, in the present study, we proceed to evaluate Ki67 performance in a risk calculator context, using IHC as the main methodology.

Tissue microarrays (TMAs) were constructed with three tissue cores per case, ensuring tumor representativeness. Immunostaining was performed using NovoLink TM Max Polymer Detection System (Leica Biosystems, Germany), as described(2). Antibody against Ki67 (M7240, Dako) was used at 1:150 dilution, overnight at room temperature. Antigen retrieval was performed with citrate buffer, 20 minutes in microwave, followed by 10 minutes colling. A lymphoma was used as positive control. Slides were analyzed by an experienced pathologist according to a semi-quantitative method. Ki67 immunostaining was categorized according to(1): 0 (<5% positive nuclei), 1 (5-10% positive nuclei), and 2 (>10% positive nuclei).

***In silico* analysis**

To unveil the most relevant CpG islands within *GSTP1* and *KLF8* promoters, TCGA Human methylation 450k array data were retrieved from Shiny Methylation Analysis Resource Tool (SMART) App website [**http://www.bioinfo-zs.com/smartapp/**](http://www.bioinfo-zs.com/smartapp/)(3). Therefore, the most significant hypermethylated CpG sites (Table S6) were selected as a region for further evaluation through qMSP. To ensure that the most promising CpGs were included in the amplification regions, the maximum possible number of significant hypermethylated CpGs were included in the primer and probes design.

**Nucleic acid extraction and bisulfite treatment**

Methylation analysis was carried out with the same paraffin blocks used for TMAs construction. Total DNA isolation was performed with the FFPE RNA/DNA Purification Plus Kit (Norgen Biotek, Thorold, Canada), according to manufacturer instructions. Bisulfite modification was carried out using 50 ng of DNA for a final concentration of 2ng/µL, with the EZ DNA Methylation-Gold Kit (REF: D5006), according to manufacturer instructions.

**Primer/probe design and quantitative Methylation Specific PCR (qMSP)**

Primers and probes (Table S7) were specifically designed based on the precise location of selected CpGs sites(4). *GSTP1* primers were already validated by us(5). QMSP reactions were run in QuantStudio 12K Flex Real-Time PCR System (Thermo Fisher, Forter, CA, USA), with β*-Actin* serving as a housekeeping gene. In brief, 1.5µL of bisulfite-modified DNA, 5µL of MasterMix Xpert Fast Probe (GRISP) with ROX, 10µM of primers and probe and sterile bi-distilled water were added to each well to a final 10µL volume (Table S8). Primer/probe annealing temperature was optimized for all genes at 60◦C. All samples were run in triplicate. No template, negative and positive controls were used as documented elsewhere(6). Run efficiency was considered valid when values varied between 90 and 100%. Results were plotted as relative methylation levels (ratio between target and housekeeping gene mean quantities multiplied by 1000, for easier tabulation).

**Statistical analysis**

Non-parametric tests were used to determine the statistical power of differences among comparable groups, using GraphPad Prism version 9.5 (GraphPad Software, La Jolla California USA). Mann-Whitney and Kruskal-Wallis tests were used for comparisons between two or multiple groups, respectively. Tumor samples were classified into low or high methylation levels based on data frequency distribution (50^th^ percentile as cutoff). Log-rank test was performed for survival analysis.

For risk calculator computation, nomograms were constructed based on multivariable logistic regression analysis, using Rstudio (R software, version 4.3.1). ROC curves evaluated models’ performance, based on area under the curve (AUC) calculations, using Rms, caTools, epicalc, and pROC packages. Youden Index was used to select the best cutoff for each graphical nomogram. For the creation of the nomograms we used the clinicopathological variables with clinical relevance: age at diagnosis (AGEcat), Grade Group (GG), clinical stage (Tstage), and PSA serum levels (PSA). For the ProstARK calculator, we include Ki67 immunoscore, the promising biomarker in study with the potential to predict progression or BCR. For the risk of death calculator, the biomarker included was *KLF8*, since it has impact on the overall survival of patients. Low- and high-risk categories were defined based on the optimal Youden’s Index, followed by risk-survival curves construction. Optimal Youden’s Index translates into a cutoff based on the linear predictor, obtaining the equivalent risk value by applying the formula: 1/(1+e^-linear predictor^). Overall, a cutoff of risk was estimated through the optimal Youden Index, 0.41 for risk of death and 0.31 for the risk of recurrence/progression.

For each analysis, p-values lower than 0.05 were considered statistically significant (*p<0.05; **p<0.01; ***p<0.001; ****p<0.0001, ns: non-significant).

Supplementary Figures

**Supplementary Figure S1
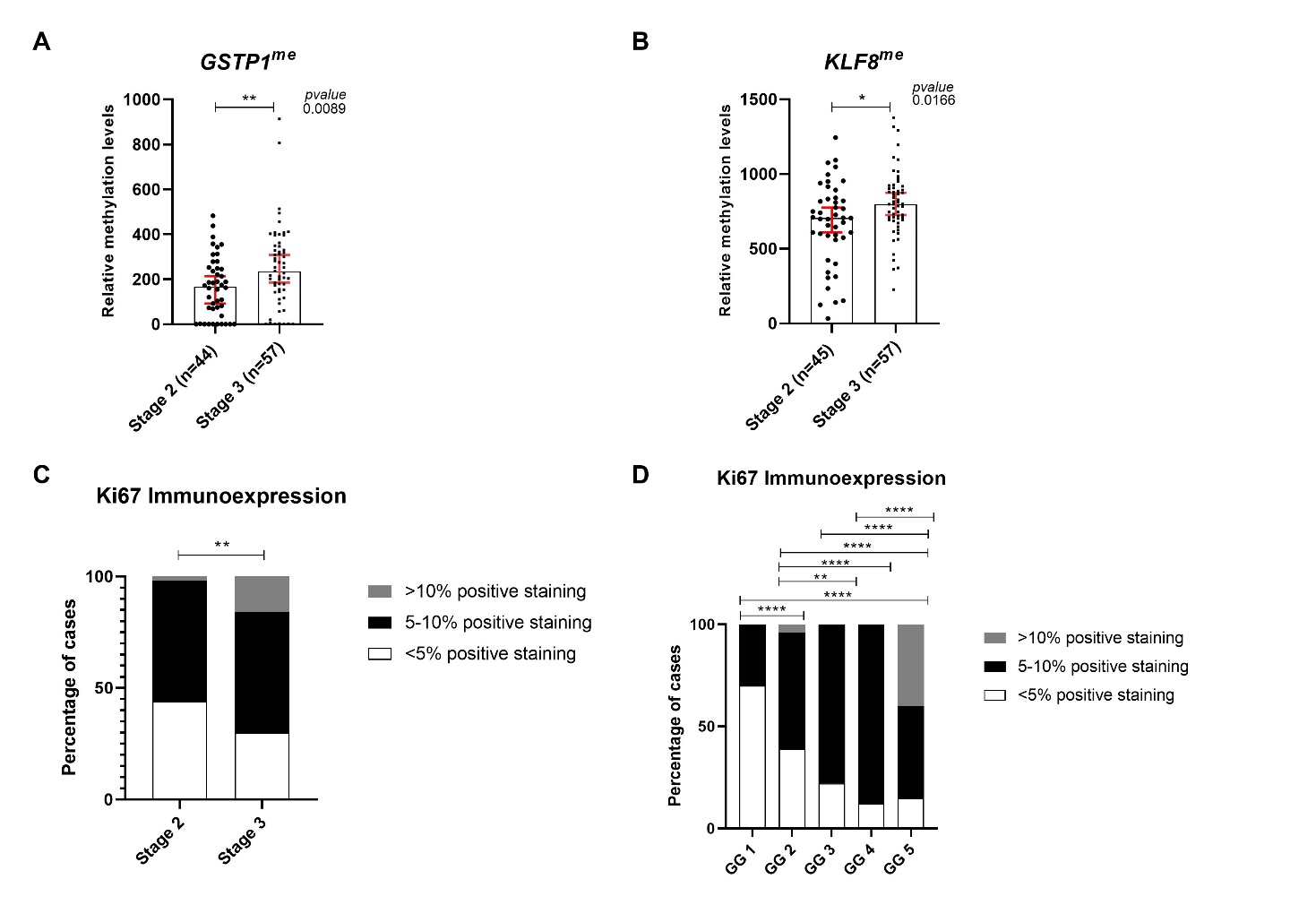
**

Supplementary Figure S1- Association of *GSTP1* and *KLF8* relative methylation levels and of Ki67 immunoexpression with clinicopathological features. A- *GSTP1^me^* and B- *KLF8^me^* levels were compared between stage II and stage III prostate tumor samples. Ki67 immunoexpression was compared between stage II and stage III tumors (C) and between different Gleason grade (GG) groups, GG1-GG5 (D). Median ranks between two groups were compared using the Mann-Whitney test and the comparisons between grade groups were performed using the Kruskal-Wallis test. *, **, *** and **** represent a p-value lower than 0.05, 0.01, 0.001 and 0.0001, respectively.

**Supplementary Figure S2**


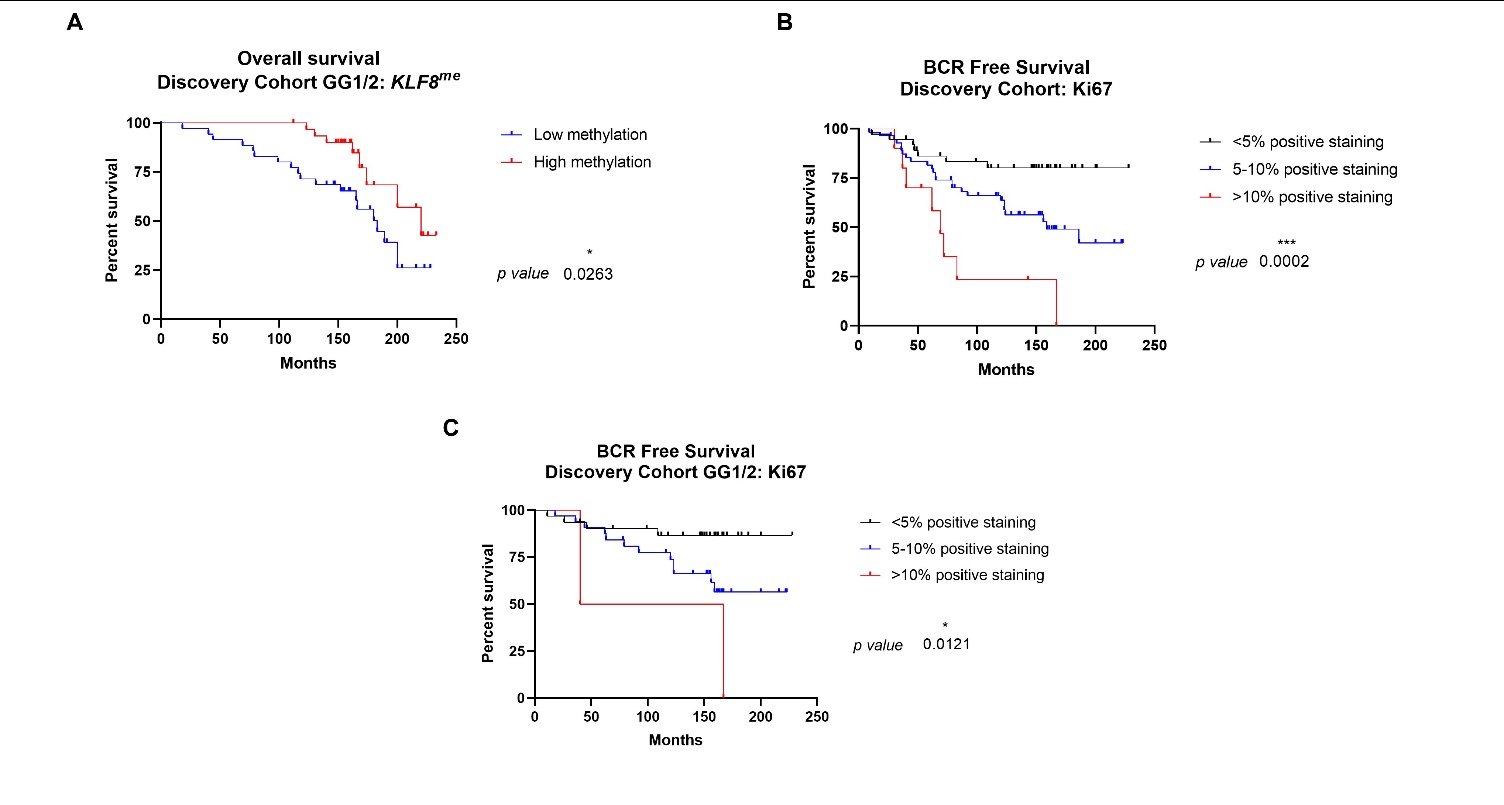


Supplementary Figure S2- Kaplan-Meier curve for the overall survival based on the methylation levels of *KLF8*, in the discovery cohort GG1/2 (A). Kaplan-Meier curves for the biochemical-recurrence free survival based on the expression of Ki67, in discovery cohort (B) and when stratified for GG1/2 (C). The Log-Rank test was used to evaluate the differences in overall survival and BCR-free survival, where * and *** represent p-values lower than 0.05 and 0.001, respectively.

**Supplementary Figure S3**


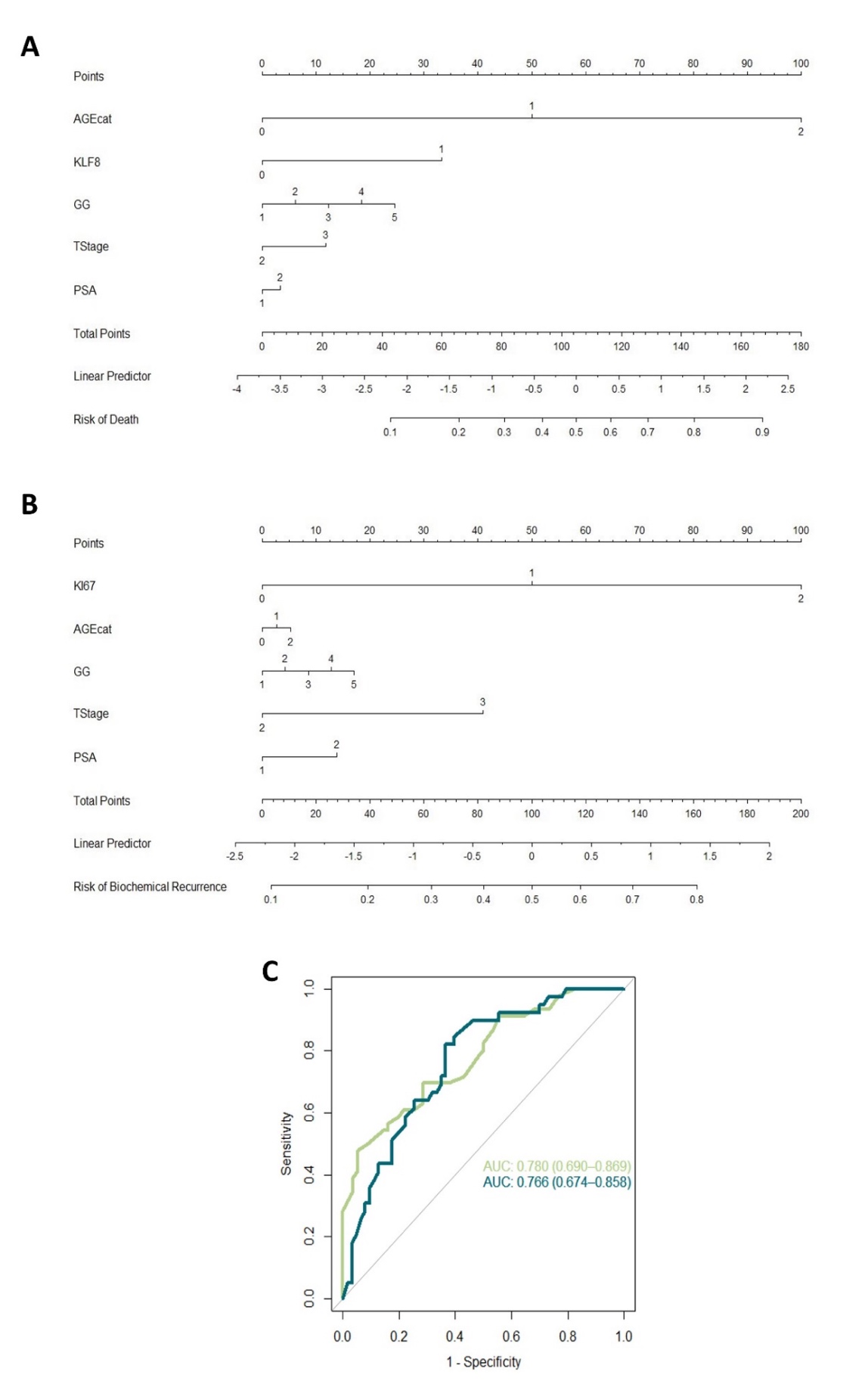


**Supplementary Figure S4**

Supplementary Figure S3- Nomogram representative of the Risk of Death (A) and the Risk of Biochemical-recurrence (B) calculators, for the Discovery Cohort, with the relevant clinicopathological variables for the risk stratification: age at diagnosis (0: ≤55, 1: 55-65, 2: >65 years), clinical stage, grade group, and PSA serum levels (1: <10ng/mL, 2: >10ng/mL). C- Roc curves to evaluate the performance of the nomogram models. *KLF8^me^* categorization- 0: high methylation levels, 1: low methylation levels. Ki67 immunoexpression categorization- 0: <5%, 1: 5-10%, 2: >10% of positive staining. Green Curve: ROC curve for the Risk of Death calculator. Blue Curve: ROC curve for the Risk of Biochemical-recurrence calculator.


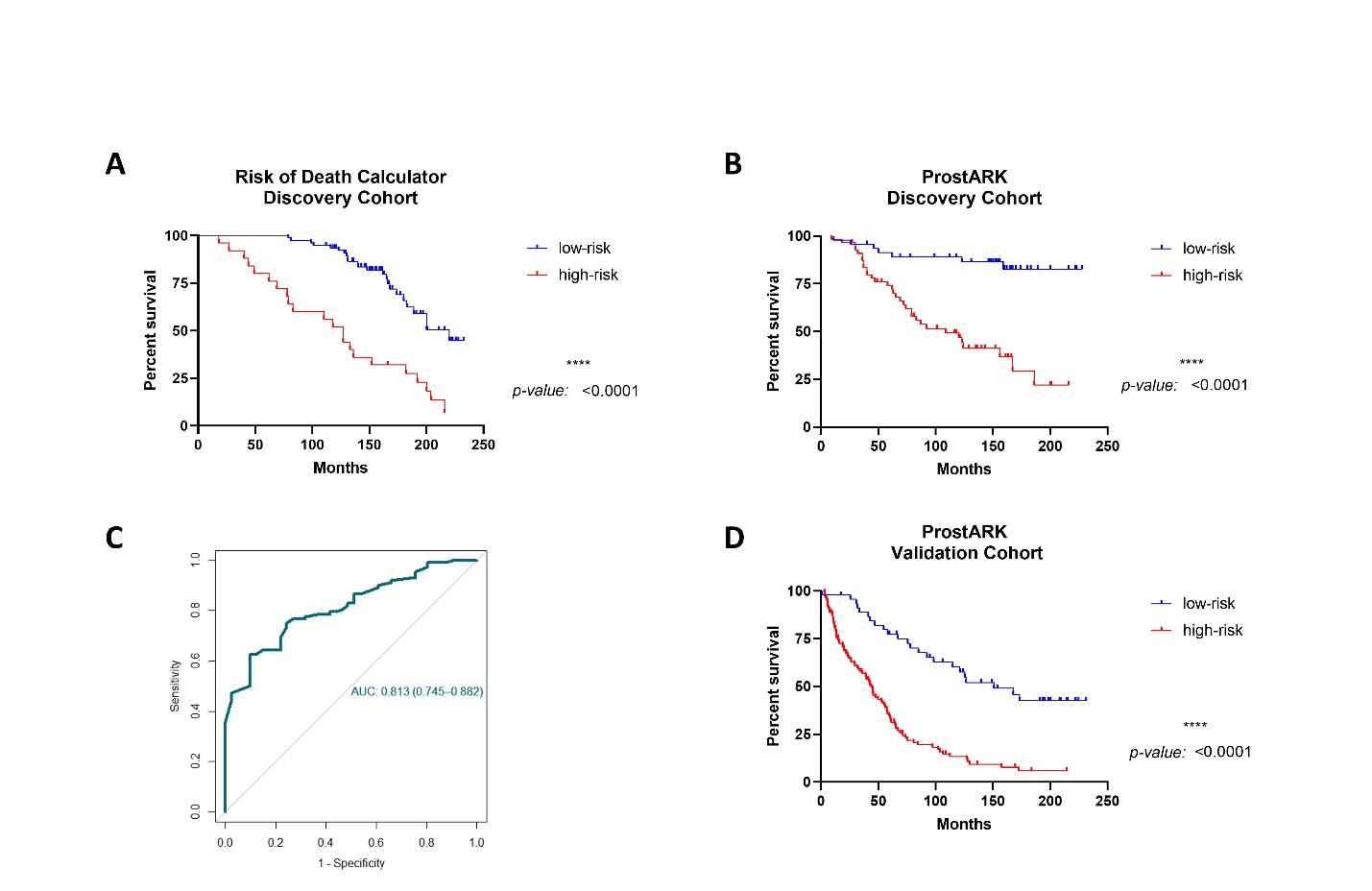


Supplementary Figure S4- A- Kaplan-Meier curve for the overall survival based on the Risk of death calculator, in the Discovery Cohort. A 0.58 cut-off for the linear predictor, translated into risk of death of 0.64. B- Kaplan-Meier curves for the biochemical-recurrence free survival based on the ProstARK calculator, in the Discovery Cohort. A -0.83 cut-off for the linear predictor, translated into recurrence/progression risk of 0.30. C- ROC curve to evaluate the performance of the ProstARK, in the Validating Cohort. D- Kaplan-Meier curves for the biochemical-recurrence free survival based on the ProstARK calculator, in Validation Cohort.

|  |  |  |  |  | **95% Confidence Interval (CI)** | |
| --- | --- | --- | --- | --- | --- | --- |
|  | **Coefficient** | **S.E.** | **Wald Z** | ***p-value*** | **Lowe Bound** | **Upper Bound** |
| **Model 1: Risk of Death Discovery Cohort GG1/2** |  |  |  |  |  |  |
| **Intercept** | -4.0594 | 1.9136 | -2.12 | 0.0339 | -7.8101 | -0.3088 |
| **AGEcat** | 1.0339 | 0.4913 | 2.10 | 0.0353 | 0.0710 | 1.9968 |
| **KLF8** | 1.7585 | 0.6456 | 2.72 | 0.0064 | 0.4932 | 3.0238 |
| **Tstage** | 0.8255 | 0.6406 | 1.29 | 0.1976 | -0.4302 | 2.0812 |
| **PSA** | -0.5032 | 0.6781 | -0.74 | 0.4581 | -1.8322 | 0.8259 |
|  |  |  |  |  |  |  |
| **Model 2: ProstARK Discovery Cohort GG1/2** |  |  |  |  |  |  |
| **Intercept** | -4.5509 | 1.9821 | -2.30 | 0.0217 | -8.4357 | -0.6661 |
| **AGEcat** | -0.1447 | 0.4912 | -0.29 | 0.7683 | -1.1075 | 0.8180 |
| **Ki67** | 1.6921 | 0.6116 | 2.77 | 0.0057 | 0.4933 | 2.8908 |
| **Tstage** | 0.5982 | 0.6454 | 0.93 | 0.3540 | -0.6666 | 1.8631 |
| **PSA** | 0.9164 | 0.6630 | 1.38 | 0.1669 | -0.3831 | 2.2160 |

Table S1- Summary of the logistic models’ representative of the Risk of Death and the Risk of BCR calculators, in the Discovery Cohort GG1/2

**Abbreviations:** S.E.- Standard Error; CI- Confidence Interval; BCR- Biochemical-recurrence; PSA- Prostate-specific Antigen

Table S2- Summary of the logistic models’ representative of the Risk of Death and the Risk of BCR calculators, in the Discovery Cohort

**Abbreviations:** S.E.- Standard Error; CI- Confidence Interval; BCR- Biochemical-recurrence; GG- Grade Group; PSA- Prostate-specific Antigen

|  |  |  |  |  | **Confidence Interval** | |
| --- | --- | --- | --- | --- | --- | --- |
|  | **Coefficient** | **S.E.** | **Wald Z** | **p-value** | **Lowe Bound** | **Upper Bound** |
| **Model 3: Risk of Death Discovery Cohort** |  |  |  |  |  |  |
| **Intercept** | -4.8703 | 1.6838 | -2.89 | 0.0038 | -8.1704 | -1.5702 |
| **AGEcat** | 1.7663 | 0.4357 | 4.05 | <0.0001 | 0.9124 | 2.6202 |
| **KLF8** | 1.1751 | 0.4889 | 2.40 | 0.0162 | 0.2168 | 2.1334 |
| **GG** | 0.2169 | 0.2024 | 1.07 | 0.2839 | -0.1798 | 0.6136 |
| **Tstage** | 0.4146 | 0.5522 | 0.75 | 0.4527 | -0.6676 | 1.4968 |
| **PSA** | 0.1172 | 0.5055 | 0.23 | 0.8167 | -0.8737 | 1.1080 |
|  |  |  |  |  |  |  |
| **Model 4: ProstARK Discovery Cohort** |  |  |  |  |  |  |
| **Intercept** | -4.5431 | 1.5207 | -2.99 | 0.0028 | -7.5237 | -1.5626 |
| **AGEcat** | 0.0594 | 0.3511 | 0.17 | 0.8657 | -0.6287 | 0.7475 |
| **Ki67** | 1.1350 | 0.4332 | 2.62 | 0.0088 | 0.2859 | 1.9841 |
| **GG** | 0.0966 | 0.2077 | 0.47 | 0.6418 | -0.3104 | 0.5036 |
| **Tstage** | 0.9295 | 0.5416 | 1.72 | 0.0861 | -0.1320 | 1.9911 |
| **PSA** | 0.3139 | 0.4790 | 0.66 | 0.5123 | -0.6250 | 1.2528 |

**Table S3-** Indicators of the calculators’ performance

|  | **SE (%)** | **95% CI** | **SP (%)** | **95% CI** | **PPV (%)** | **NPV (%)** | **AC (%)** | **AUC** | **95% CI** |
| --- | --- | --- | --- | --- | --- | --- | --- | --- | --- |
| **ProstARK, Discovery Cohort GG1/2** | 66.67 | 43.75 - 83.72 | 79.17 | 65.74 - 88.27 | 55 | 86 | 75.76 | 0.75 | 0.62 - 0.89 |
| **Risk of Death Calculator, Discovery Cohort GG1/2** | 75.00 | 55.10 - 88.00 | 66.67 | 50.98 - 79.37 | 58 | 81 | 69.84 | 0.76 | 0.64 - 0.89 |
| **ProstARK, Discovery Cohort** | 82.05 | 67.33 - 91.02 | 63.49 | 51.15 - 74.28 | 58 | 85 | 70.59 | 0.77 | 0.67 - 0.86 |
| **Risk of Death Calculator, Discovery Cohort** | 47.83 | 34.12 - 61.86 | 94.64 | 85.39 - 98.54 | 92 | 69 | 74.51 | 0.78 | 0.69 - 0.87 |

**Abbreviations:** SE- Sensitivity; CI- Confidence Interval; SP- Specificity; PPV- Positive Predictive Value; NPV- Negative Predictive Value; AC- Accuracy; AUC- Area under the curve

Table S4- Detailed clinicopathological data of PCa patients.

|  | **Discovery Cohort** | **Discovery Cohort GG1/2** |
| --- | --- | --- |
| **Clinicopathological Characteristics** | **Mean ± standard deviation, total n=103** | **Mean ± standard deviation, total n=66** |
| **Age (Years)** | 63.3 ± 6.03 | 63.4 ± 5.67 |
| **Clinicopathological Characteristics** | **Patient Numbers n (%), total n=103** | **Patient Numbers n (%), total n=66** |
| **Age (Categorized)**  ≤55 years  55-65  >65 |  |  |
|  | 12 (11.65%) | 7 (10.61%) |
|  | 53 (51.46%) | 33 (50.00%) |
|  | 38 (36.89%) | 26 (39.39%) |
| **Serum PSA concentration (ng/mL)**  <10  10-20  >20 |  |  |
|  | 66 (64.08%) | 48 (72.73%) |
|  | 36 (34.95%) | 18 (27.27%) |
|  | 1 (0.97%) | N/A |
| **ISUP Grade Group**  1  2  3  4  5 |  |  |
|  | 20 (19.42%) | 20 (30.30%) |
|  | 46 (44.66%) | 46 (69.70%) |
|  | 9 (8.74%) | N/A |
|  | 8 (7.77%) | N/A |
|  | 20 (19.42%) | N/A |
| **Clinical Stage**  pTt2a  pT2b  pT2c  pT3a  pT3b  pT4 |  |  |
|  | 1 (0.97%) | 1 (1.52%) |
|  | 2 (1.94%) | 2 (3.03%) |
|  | 42 (40.78%) | 39 (59.09%) |
|  | 43 (41.75%) | 20 (30.30%) |
|  | 14 (13.59%) | 4 (6.06%) |
|  | 1 (0.97%) | N/A |
| **Biochemical Recurrence** | 35 (33.98%) | 18 (27.27%) |
| **Clinical Recurrence** | 18 (17.48%) | 3 (4.54%) |
| **Deaths** | 46 (44.66%) | 27 (40.91%) |

**Abbreviations:** N/A- Non applicable

Table S5- Detailed clinicopathological data of a series of prostate cancer biopsies, previously described in our group (1).

|  | **Validation Cohort** | **Validation Cohort GG1/2** |
| --- | --- | --- |
| **Clinicopathological Characteristics** | **Mean ± standard deviation, total n=153** | **Mean ± standard deviation, total n=42** |
| **Age (Years)** | 71.7 ± 6.78 | 69.2 ± 5.80 |
| **Clinicopathological Characteristics** | **Patient Numbers n (%), total n=153** | **Patient Numbers n (%), total n=42** |
| **Age (Categorized- years)** |  |  |
| ≤55 | 2 (1.31%) | 1 (2.38%) |
| 55-65 | 27 (17.65%) | 7 (16.67%) |
| >65 | 124 (81.04%) | 34 (80.95%) |
| **Serum PSA concentration (ng/mL)** |  |  |
| ≤10 | 18 (11.77%) | 12 (28.58%) |
| 10-20 | 29 (18.95%) | 15 (35.71%) |
| >20 | 106 (69.28%) | 15 (35.71%) |
| **ISUP Grade Group** |  |  |
| 1 | 17 (11.11%) | 17 (40.48%) |
| 2 | 25 (16.34%) | 25 (59.52%) |
| 3 | 36 (23.53%) | N/A |
| 4 | 60 (39.22%) | N/A |
| 5 | 15 (9.80%) | N/A |
| **Clinical Stage** |  |  |
| cT1 | 2 (1.31%) | 1 (2.38%) |
| cT2 | 47 (30.72%) | 29 (69.05%) |
| cT3 | 59 (38.56%) | 11 (26.19%) |
| cT4 | 45 (29.41%) | 1 (2.38%) |
| **Biochemical Recurrence** | 112 (73.20%) | 19 (45.24%) |
| **Deaths** | 122 (79.74%) | 23 (54.76%) |

**Abbreviations:** N/A- Non applicable

Table S6- In silico analysis for the most relevant methylated CpGs in the promoter region of the *GSTP1* and *KLF8* genes

| **Gene** | **Number of Hypermethylated CpGs** |
| --- | --- |
| *GSTP1* | 6 |
| *KLF8* | 3 |

Table S7- Primers and Probe sequences with respective fluorochrome and quencher

| **Gene** | **Sequence** | |
| --- | --- | --- |
| ***Β-Actin*** | **Primers** | F – 5’ TGGTGATGGAGGAGGTTTAGTAAGT 3’ |
|  |  | R – 5’ AACCAATAAAACCTACTCCTCCCTTAA 3’ |
|  | **Probe** | 5’ Cy5 – ACCACCACCCAACACACAATAACAAACACA – BHQ 3’ |
| ***GSTP1*** | **Primers** | F – 5’ AGTTGCGCGGCGATTTC 3’ |
|  |  | R – 5’ GCCCCAATACTAAATCACGACG 3’ |
|  | **Probe** | 5’ FAM – CGGTCGACGTTCGGGGTGTAGCG – TAMRA 3’ |
| ***KLF8*** | **Primers** | F – 5’ GGCGCGAGGTATTTCGTG 3’ |
|  |  | R – 5’ CCCCAAAACAAAAACTTCAACG 3’ |
|  | **Probe** | 5’ FAM – TTCGTTTTAGAGGATTCGTACGAGTTGTTG – BHQ1 3’ |

Table S8- Conditions of qMSP for each gene

| **Gene** | **Primers, volume (µL), F+R** | **Probe, volume (µL)** | **Annealing Temperature (**^◦^**C)** |
| --- | --- | --- | --- |
| **β-*Actin*** | 0.5 | 0.05 | 60^◦^C |
| ***GSTP1*** | 0.3 | 0.05 | 60^◦^C |
| ***KLF8*** | 0.3 | 0.05 | 60^◦^C |

**Supplementary Information 1**

A nomogram is a graphical calculator device based on logistic regression models. This tool is widely used in clinical practice for patient outcome prediction, helping clinicians make treatment decisions(7, 8). The first step to creating a nomogram is creating a logistic regression model that fits better with our data. All models and consequently, all nomograms were created using the R software with the following codes. Afterward, ROC curves were used to evaluate the performance of our models. The rms, caTools, epicalc, and pROC packages were installed for these analyses.

Example 1: All variables are categorized. This example was used to perform a nomogram using a logistic regression model for overall survival with all variables.

>Model1<-lrm(VitalStatus~AGEcat+KLF8+TStage+GG+KI67+PSA,data=Geral)

>ddist<-datadist(Geral)

>options(datadist='ddist')

>nom1<-nomogram(Model1,fun=plogis,funlabel = "Risk of Death")

>plot(nom)

As for ROC curves, the following codes were applied:

>Model1<-glm(formula=VitalStatus~AGEcat+KLF8+TStage+GG+KI67+PSA, family=binomial(link = "logit"),data=Geral)

>summary(Model1)

>logistic.display(Model1)

>par(pty="s")

>ROCcurve1<-roc(Geral$VitalStatus~Model1$fitted, plot=TRUE,legacy.axes=TRUE,print.auc=TRUE,ci=TRUE,lwd=4,col="#b5d08d")

**References of Additional File 1**

1. Lobo J, Rodrigues Â, Antunes L, Graça I, Ramalho-Carvalho J, Vieira FQ, et al. High immunoexpression of Ki67, EZH2, and SMYD3 in diagnostic prostate biopsies independently predicts outcome in patients with prostate cancer. Urol Oncol. 2018;36(4):161.e7-.e17.

2. Lima T, Macedo-Silva C, Felizardo D, Fraga J, Carneiro I, Jerónimo C, et al. Gal-3 Protein Expression and Localization in Prostate Tumours. Curr Oncol. 2023;30(3):2729-42.

3. Li Y, Ge D, Lu C. The SMART App: an interactive web application for comprehensive DNA methylation analysis and visualization. Epigenetics Chromatin. 2019;12(1):71.

4. Lobo J, Constâncio V, Guimarães-Teixeira C, Leite-Silva P, Miranda-Gonçalves V, Sequeira JP, et al. Promoter methylation of DNA homologous recombination genes is predictive of the responsiveness to PARP inhibitor treatment in testicular germ cell tumors. Mol Oncol. 2021;15(4):846-65.

5. Jerónimo C, Usadel H, Henrique R, Oliveira J, Lopes C, Nelson WG, et al. Quantitation of GSTP1 methylation in non-neoplastic prostatic tissue and organ-confined prostate adenocarcinoma. J Natl Cancer Inst. 2001;93(22):1747-52.

6. Macedo-Silva C, Constâncio V, Miranda-Gonçalves V, Leite-Silva P, Salta S, Lobo J, et al. DNA methylation biomarkers accurately detect esophageal cancer prior and post neoadjuvant chemoradiation. Cancer Med. 2023;12(7):8777-88.

7. Raghav K, Hwang H, Jácome AA, Bhang E, Willett A, Huey RW, et al. Development and Validation of a Novel Nomogram for Individualized Prediction of Survival in Cancer of Unknown Primary. Clin Cancer Res. 2021;27(12):3414-21.

8. Zhang Z, Kattan MW. Drawing Nomograms with R: applications to categorical outcome and survival data. Ann Transl Med. 2017;5(10):211.
